# High-Throughput In Vivo Analysis of Gene Expression in Caenorhabditis elegans

**DOI:** 10.1371/journal.pbio.0050237

**Published:** 2007-09-11

**Authors:** Rebecca Hunt-Newbury, Ryan Viveiros, Robert Johnsen, Allan Mah, Dina Anastas, Lily Fang, Erin Halfnight, David Lee, John Lin, Adam Lorch, Sheldon McKay, H. Mark Okada, Jie Pan, Ana K Schulz, Domena Tu, Kim Wong, Z Zhao, Andrey Alexeyenko, Thomas Burglin, Eric Sonnhammer, Ralf Schnabel, Steven J Jones, Marco A Marra, David L Baillie, Donald G Moerman

**Affiliations:** 1 Department of Zoology, University of British Columbia, Vancouver, British Columbia, Canada; 2 Michael Smith Laboratories, University of British Columbia, Vancouver, British Columbia, Canada; 3 Department of Molecular Biology and Biochemistry, Simon Fraser University, Burnaby, British Columbia, Canada; 4 Genome Sciences Centre, British Columbia Cancer Agency, Vancouver, British Columbia, Canada; 5 Institut für Genetik, Technische Universität Braunschweig, Braunschweig, Germany; 6 Center for Genomics and Bioinformatics, Karolinska Institutet, Stockholm, Sweden; 7 Department of Biosciences, Karolinska Institutet, Huddinge, Sweden; Wellcome Trust Sanger Institute, United Kingdom

## Abstract

Using DNA sequences 5′ to open reading frames, we have constructed green fluorescent protein (GFP) fusions and generated spatial and temporal tissue expression profiles for 1,886 specific genes in the nematode Caenorhabditis elegans. This effort encompasses about 10% of all genes identified in this organism. GFP-expressing wild-type animals were analyzed at each stage of development from embryo to adult. We have identified 5′ DNA regions regulating expression at all developmental stages and in 38 different cell and tissue types in this organism. Among the regulatory regions identified are sequences that regulate expression in all cells, in specific tissues, in combinations of tissues, and in single cells. Most of the genes we have examined in C. elegans have human orthologs. All the images and expression pattern data generated by this project are available at WormAtlas (http://gfpweb.aecom.yu.edu/index) and through WormBase (http://www.wormbase.org).

## Author Summary

Knowing where a protein is expressed provides an important clue about its potential function. As critical as this information is, we have complete developmental expression profiles for only a small fraction of all genes expressed in any metazoan. Here, we have generated spatial and temporal tissue expression profiles for 10% of all genes in the nematode Caenorhabditis elegans. Worms expressing putative gene regulatory elements fused with green fluorescent protein were analyzed at each stage of development from embryo to adult. Among the regulatory regions identified are sequences that regulate expression in all cells, in specific tissues, in combinations of tissues, and in single cells. Most of the genes we have examined in C. elegans have human orthologs. Our analysis of complex expression patterns for so many genes may not only facilitate functional analysis in *C. elegans,* but also create a foundation for decoding the informational hierarchies governing gene expression in all organisms.

## Abstract 3

Using DNA sequences 5' to open reading frames, the authors construct green fluorescent protein fusions and generate spatial and temporal tissue expression profiles for 10% of all genes in the nematode *Caenorhabditis elegans*.

## Introduction

Determining when and where genes are expressed is often key to determining their function. Although expression profiling of genes using Serial Analysis of Gene Expression (SAGE) and microarrays is now routine, we still have complete developmental expression profiles for only a small fraction of all genes expressed in any metazoan. The spatial resolution of these two techniques is limited unless purified cell populations can be isolated in sufficient abundance to provide the necessary RNA (for examples, see [1–3]). How then do we gain expression information on the thousands of human genes that are still largely uncharacterized? One approach is to use high-throughput RNA in situ hybridization as has recently been done for brain tissue in the mouse [4]. In this study, 20,000 genes were assayed in the adult male mouse brain, and their distribution in many cases was resolved to the level of a single cell. Another complementary approach involves employing green fluorescent protein (GFP) [5] as a marker to monitor gene expression in a specific cell or tissue. The GenSAT project [6] uses Bacterial Artificial Chromosomes (BACs) with GFP-marked genes in transgenic mice to monitor tissue and cell expression. About 2,000 gene expression patterns are described at the GenSAT site (http://www.gensat.org/). Because gene functions were largely maintained during evolution, yet another possible approach is to first study orthologs of these genes in less complex organisms. Knowing what tissue or cell type expresses a particular gene in a simpler system such as Caenorhabditis elegans or Drosophila melanogaster could help drive the analysis of this gene in a more complex tissue or organ system, as is found in mice and humans. In *Drosophila,* a large-scale in situ hybridization study has now documented the expression pattern of close to 3,000 genes in the developing embryo ([7]; http://www.fruitfly.org/cgi-bin/ex/insitu.pl). The goal of our study was to characterize the temporal and spatial expression pattern of human orthologs in the nematode C. elegans down to the resolution of a single cell. Specifically, we determined the expression profile of individual genes throughout the whole organism and across all life stages. Independent of the biomedical aspects of our approach, the analysis of complex expression patterns of many genes may not only facilitate functional analysis in C. elegans and other organisms, but also create a foundation for decoding the informational hierarchies governing gene expression.

C. elegans has several advantages as a venue for expression studies at this resolution. The main advantages are that it is one of the simplest multicellular organisms with a complete genome sequence available [8] and a completely documented cell lineage [9,10]. In addition, the small size, transparency, and limited cell number of the worm allow for the easy observation of many complex cellular and developmental processes that are difficult to observe in higher eukaryotes, and morphogenesis can be observed at the level of a single cell [11].

Besides ourselves, only two groups have attempted large-scale expression profiling in C. elegans at this resolution. Hope and colleagues in the past have used lacZ reporters and currently are using the newly developed “promoterome” to characterize gene expression [12–14]. Another approach, developed by Yuji Kohara's group in Japan, uses in situ hybridization to fixed animals at different developmental stages (http://nematode.lab.nig.ac.jp). Our approach was to examine expression in living animals transformed with GFP fused to DNA 5′ of genes with human orthologs. For gene fusion and amplification, we used “PCR stitching” [15], which proved to be a fast, efficient, and economical method for obtaining such constructs, and we have demonstrated that the method is scalable [1]. Because of the relatively small intergenic regions in the C. elegans genome, typically less than 3 kb, PCR stitching did not have to be done over large intervals. These small intergenic intervals illustrate yet another advantage of doing this type of study in the nematode. This is a key advantage that sets our project apart from previous high-throughput expression projects done in other organisms. Our overall approach takes advantage of the transparency of the nematode and allows us to visualize gene expression in vivo, in real time, in a living animal. This method allowed us to determine the temporal and spatial distribution of the expressed GFP in close to 10% (1,886) of all genes identified in this organism.

## Results

### Determining the Temporal and Spatial Expression of C. elegans Genes

Expression patterns analyzed for the 1,886 genes in this study were primarily, but not exclusively, from nematode orthologs of human genes (>80%). Our target genes were drawn from nematode–human ortholog groups in the InParanoid database [16] (http://inparanoid.sbc.su.se), selecting primarily genes for which no function is known. To analyze the in vivo spatial and temporal expression profiles of thousands of genes, we needed a high-throughput approach for GFP fusion constructs. GFP has been shown to be an effective cell marker in C. elegans [5,17], and because of the need for cost-effectiveness and scalability, we chose to use the promotor::GFP fusion technique “PCR stitching” [15]. The 5′ regulatory regions examined in this study extend a maximum of 3 kb upstream of the predicted ATG initiator site for a targeted gene. Most often, an upstream gene was nearer than 3 kb and we did not extend our analysis into or past this adjacent gene. As a benchmark and internal control, 10% of our analysis included genes with expression annotation in WormBase. We used half of these benchmark genes and found that 80% of our observations on expression matched the annotated expression patterns. For another 10% of the benchmark genes, we found some overlap, and for about 10%, we found little or no agreement with expression patterns compiled at WormBase. (Table S1 **)**.

**Figure 1:**
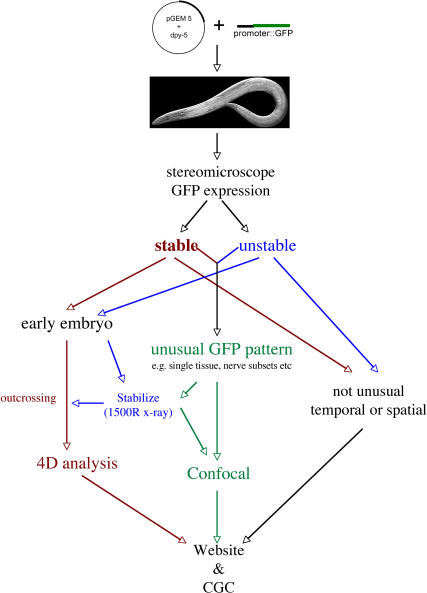
Analysis Pipeline for GFP Expressing Strains Strains with nonubiquitous embryonic expression prior to 2-fold stage were stabilized for 4-D analysis, and all postembryonic strains were briefly assessed for their expression pattern complexity and then assigned to either the confocal microscope or the stereomicroscope for detailed observations. Once the expression analysis was complete, the data were sent to the public domain, and the strains were sent to the CGC (Caenorhabditis Genetics Center).

Transformants carrying GFP fusions were subject to detailed in vivo analysis as outlined in Figure 1. We have observed GFP expression for 1,886 genes. Because we only sampled 10% of the genes in this organism, we wanted to ensure that specific functional categories were not overrepresented in our dataset. We used Gene Ontology (GO) annotation to examine the genes in our set relative to the whole genome and found that the representation of most functional groups reflected their frequency within the genome (Figure S1). Besides the genes for which we detected expression, there were another 516 genes for which we did not detect any expression (see Discussion). At present, only 15% of the strains exhibiting expression are in stable strains (possibly chromosomal integrants). As is usual for microinjected transgenes, most strains carry unincorporated concatamer arrays, and we detected mosaicism in many of these strains. To compensate for this mosaicism, and to ensure that we did not miss expressing cells, at least 20 replicates were analyzed for each developmental stage. Only GFP-expressing cells and tissues that showed consistent expression in 50% of the animals at any given developmental stage were recorded.

**Figure 2:**
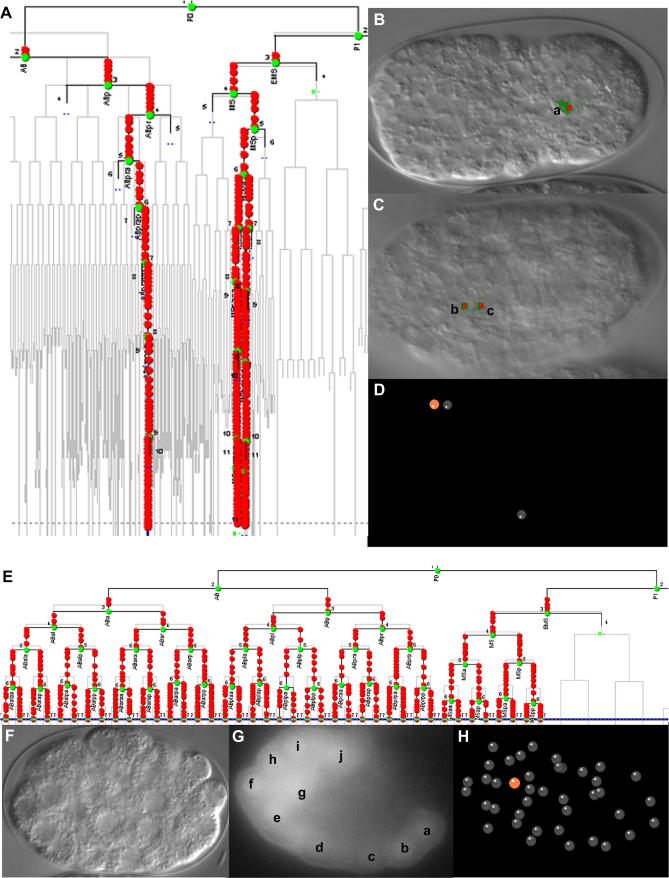
Embryonic Cell Lineages of C45G9.13 and ZK637.11 (A) and (E) display the embryonic cell lineages of C45G9.13 and ZK637.11, respectively. (B) and (C) are Nomarski differential contrast microscopy images of C45G9.13 with a GFP overlap. The view is from the ventral side with anterior to the left of the image. The highlighted cells are a, ABprappppa; b, M5; and c, Mspapaapa. (F) and (G) show Nomarski images of ZK637.11 with ventral to the bottom and anterior to the left. The cells marked in (G) are a, ABprppa; b, ABprpap; c, MSppa; d, MSpaa; e, ABalaap; f, ABalpaa; g, ABalapp; h, ABalppa; I, ABplaap; and j, ABplapp. (D) and (H) are 3-D stick and ball models of the nuclei of the expressing cells of C45G9.13 and ZK637.11, respectively. In both panels, anterior is to the left and ventral is on the bottom. In (D), the orange ball represents M5: note that the embryo has been rotated 90° to the right compared to the above Nomarski image. In (H), the orange ball corresponds to ABalapp.

Two subclasses of expressing strains were further analyzed: (1) those with rare or complex expression patterns and (2) those that showed embryonic expression before the comma stage of embryogenesis. In the former case, the strains underwent their final analysis via 2-D and 3-D imaging on a confocal microscope before being submitted to the public Web site. In the latter case, the embryonic strains were first integrated (see Materials and Methods) and then recorded during development using a four-dimensional (4-D) microscope system (multifocal, time-lapse video recording system) developed for the purpose of tracking embryonic cell identities and movements [18,19]. Since the cell lineage of C. elegans is invariant [10], we could use these recordings in conjunction with Simi BioCell software [19] to retrace the cell lineages and determine the identity of the cells expressing GFP. This has resulted in 95 embryonic recordings, two examples of which are illustrated in Figure 2. In the first, pC45G9.13 (Figure 2A–2D), expression is initially detected in three cells, ABprappppa, M5, and MSpapaapa, but later expands to include several other cells. In the second example, pZK637.11 (Figure 2E–2H), expression is detected early during embryogenesis, and includes the AB and MS lineages. At present, only a portion (10%) of the embryonic recordings have been completely analyzed and the lineage of all GFP expressing cells determined.

**Figure 3:**
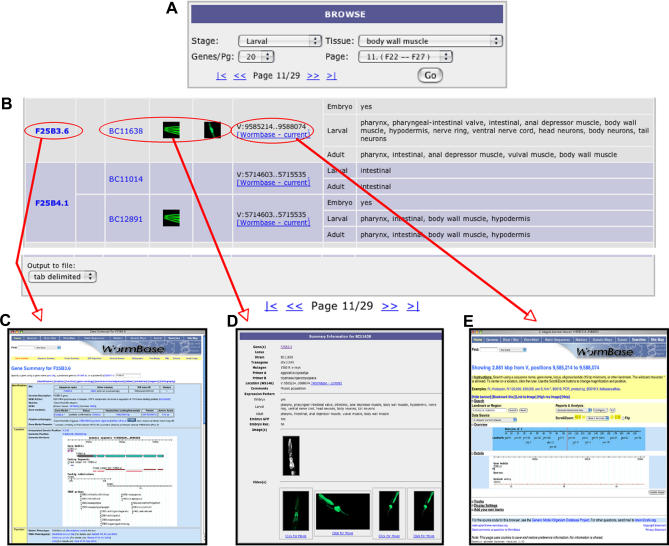
The Browse Page The Browse page initially lists all genes (B) with the relevant information as text or links, but is searchable (A) by developmental stage or a specific tissue. The links lead to (C) the Wormbase Gene Summary, (D) a comprehensive summary page specific to that strain, and (E) the Wormbase location map.

**Figure 4:**
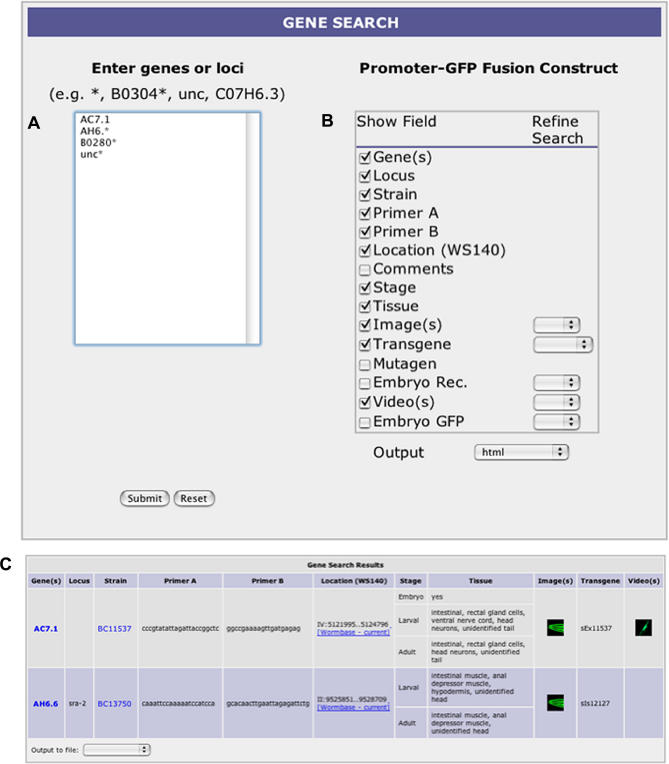
The Gene Search Page and Search Results The Search page (A) and (B) allows the user to search the dataset with a combination of gene names and data types. The user can enter (A) specific gene names or use an asterisk (*) to represent all genes and then choose (B) the list of data types representing specific information about the gene(s) of interest. The logic for the Field column is OR, and the results (C) will display columns for the data types. The Refine Search column (B) operates under AND logic for the selected field, and OR logic between fields, e.g., selecting “stable” transgene and “yes” Images will output all genes that are either stable or have an associated image. Thus the genes displayed are limited only to those that evaluate to True for a selected field.

The data from this project are publicly available at WormBase and interactively at WormAtlas (http://gfpweb.aecom.yu.edu/index). All strains are available from the *Caenorhabditis* Genetics Center (http://www.cbs.umn.edu/CGC/CGChomepage.htm) (currently, strain requests go through R. Johnsen [bjohnsen@gene.mbb.sfu.ca]). Our Web site (http://gfpweb.aecom.yu.edu/index) provides the user with two formats for accessing the data: (1) a Browse page (Figure 3) to display all strains and data, with a search option for stage or tissue, and (2) a Gene Search page (Figure 4) that enables the user to recover selected information on specific genes of interest, or identify a subset of genes from the entire dataset (e.g., show genes that are unc and have associated movies). Each gene displayed has links through the gene name and location to WormBase's Gene Summary and mapping pages (Figures 3C and 4C). The strain name has a link to a comprehensive summary page containing all data relevant to that strain (Figure 3D). Along with the data present on the initial search readout page, other information included are the primers used to amplify the promoter, whether the strain is stabilized, and links to additional images of the strain.

### GFP Expression Observed in Every Major Tissue and Cell Type

**Figure 5:**
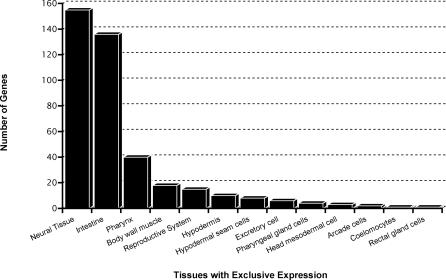
A Categorization by Tissue, Summarizing the Influence of the 389 5′ DNA Sequences that Drive Expression in a Single Tissue or Cell Type

**Figure 6:**
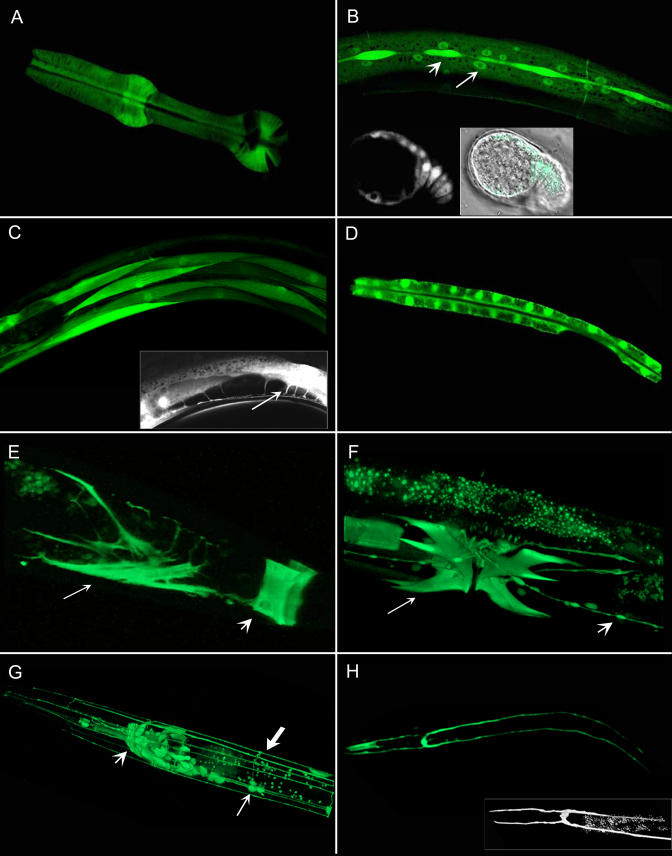
A Portrait of C. elegans General Tissue Expression Patterns, Driven by 5′ DNA::GFP Constructs (A) Pharynx—gene C32F10.8. (B) Hypodermis (long arrow) and seam cell (short arrow)—gene F25H2.1 (inset: C29E4.8—embryonic hypodermis). (C) Body wall muscle (arrow points to muscle arm)—gene F27D9.5 (inset: W01A11.1—muscle innervation). (D) Gut—gene Y102A11A.2. (E) Stomatointestinal muscle (long arrow) and anal depressor muscle (short arrow)—gene D1081.2. (F) Vulval muscle (long arrow) and ventral nerve cord (short arrow)—gene Y32H12A.5. (G) Neural (nerve ring: short arrow, ventral nerve cord: long thin arrow, and dorsal nerve cord: thick arrow)—gene C13F10.4. (H) Excretory cell—gene F32F2.1 (inset: F48E8.3) (intersects with WormBase annotation).

A survey of temporal and spatial GFP expression patterns for all 1,886 genes is shown in Table 1 and Figure 5, and some illustrative examples in different tissues are displayed in Figure 6. We have detected GFP at all developmental stages and have identified expressed GFP in all major tissues except the germinal gonad. Most GFP fusions express across all developmental stages with 1,781 (95%) showing expression in adults, 1,835 (97%) in larval animals, and 1,556 (83%) expressing during embryogenesis. A majority of the 5′ regulatory DNA sequences examined drive GFP expression in the nervous system (63%), the intestine (63%), the pharynx (40%), and the body-wall muscle (32%)** (** Table 1 **)**. Subsets of cells and tissues within these broad categories are also delineated; we have observed GFP expression specific to the nerve ring, sensory neurons, ventral nerve cord, pharynx, seam cells, the excretory canal and excretory gland cells, the spermatheca, and coelomocytes, to list a few.

**Table 1:**
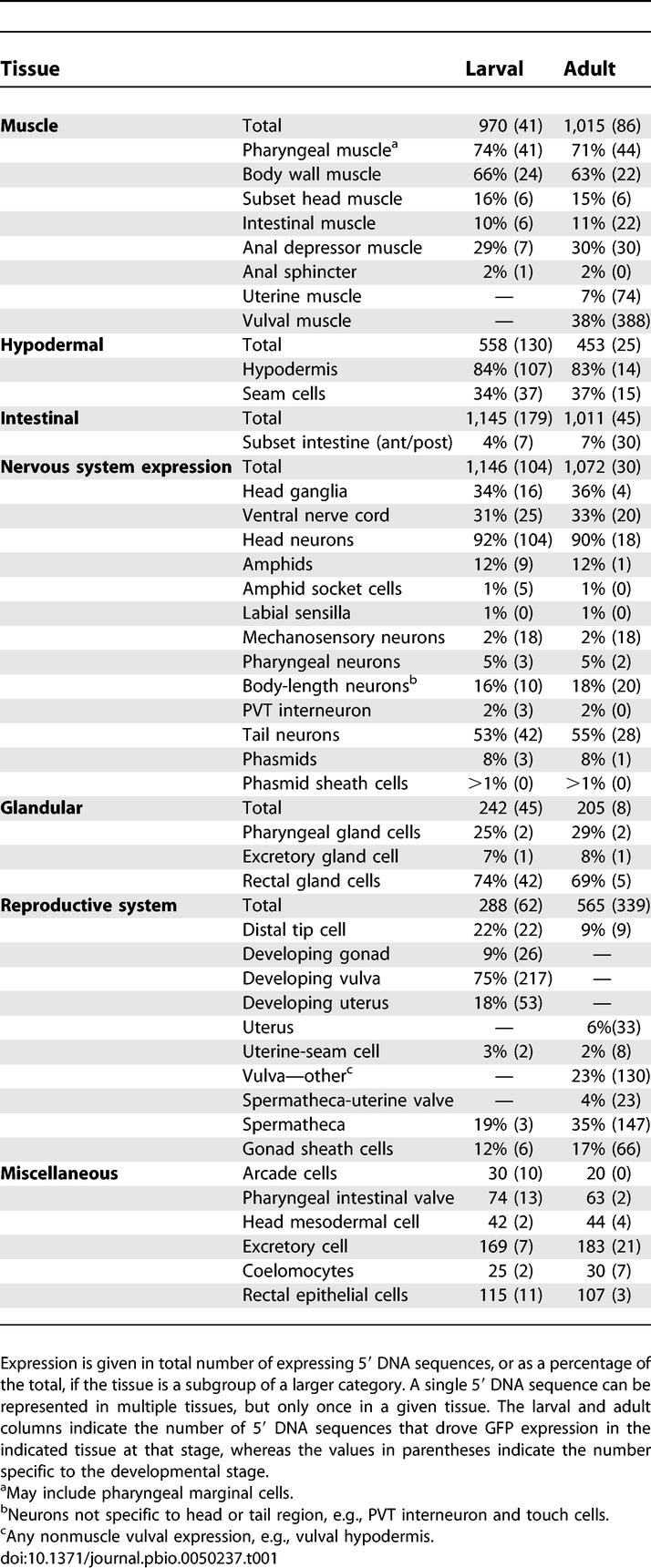
Spatial and Temporal Expression of the 5′ DNA::GFP Fusions for the 1,886 Expressing Sequences

Over the course of our analysis, we observed GFP expression in 38 tissues and cell types throughout all developmental stages: embryo, larval (L1–L4) and adult (Figure 6; Table 1). We observed many examples of temporal expression stability and examples where the expression pattern changed during development. For example, pF26F4.6::GFP exhibited hypodermal expression during the larval stages, but no GFP was detectable in adult hypodermis; pY61A9LA.10::GFP showed intestinal and neural expression during early developmental stages, whereas adults lacked any GFP expression at all. Conversely, we observed cases where GFP expression was turned on later in development, as in the case of pF11F1.1::GFP, where no GFP was detected until the animals matured to adults, at which point hypodermal and intestinal expression were observed. Examples of changing patterns of expression formed a minority of our dataset. This, in some respects, was to be expected because we used the enhanced form of GFP (EGFP), which has a long half-life. Early expressed embryonic GFP could persist through the 14 h (22 °C) duration of embryogenesis [20] and possibly past hatching. Similarly, GFP expressed during larval development may persist in adult tissues. Also, embryonic expression was never detected earlier than the 50–100 cell stage of embryogenesis, possibly a consequence of our inability to detect maternal RNA contributions to the developing embryo [21] (see Discussion).

Although the concatameric arrays may have led to germline silencing in the gonad [21], they may also have contributed to increasing the sensitivity of detecting an expression signal in other tissues. As described in Materials and Methods, each array has several copies of the fusion GFP construct. Several of these GFP fusions can express simultaneously in a particular cell. As a test of the sensitivity of GFP fusions, we used them to see if we could detect expression from genes with low numbers of SAGE tags. Specifically, we were able to detect a GFP signal for 232 genes that only had a single tag in either the embryo or one of the following tissues: neurons, hypodermis, intestine, or muscle. In each case, GFP expression was detected in the tissue for which only a single SAGE tag had been recorded (Table S2). The SAGE data can be viewed at http://tock.bcgsc.ca/cgi-bin/sage170 and WormBase. The source of this material is from different developmental stages and different purified tissue and cell populations during early development ([1] and unpublished data).

To measure the reliability and accuracy of the reporter expression patterns described in this study, we took further advantage of the existing SAGE data for specific tissues. We have compared the intersects between our GFP expression patterns for muscle, gut, and the nervous system against SAGE data for stage-specific purified cells populations for each of these tissues. In each case, we can identify about 70% of GFP reporter genes in the corresponding SAGE library (e.g., genes for 71% of GFP reporters expressed in muscle are detected in the muscle SAGE library; unpublished data). Considering that the SAGE libraries are limited to embryonic tissue only and that half of the SAGE tags are present in single copies, we believe this is a reasonable validation of the GFP reporter expression patterns observed.

### Most 5′ Regulatory Sequences Drive Expression in Multiple Tissues

**Figure 7:**
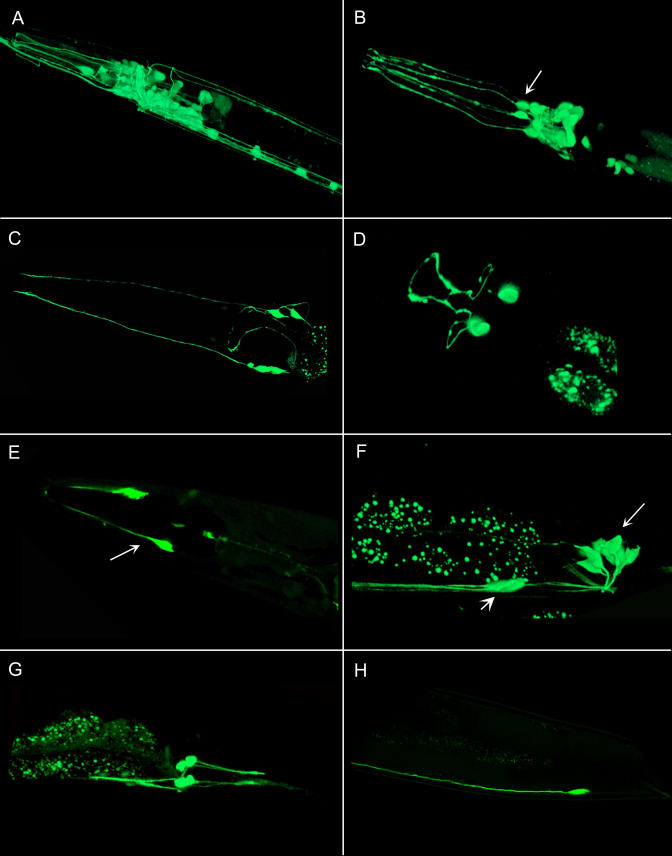
A Portrait of C. elegans Neural Tissue Expression Patterns, Driven by 5′ DNA::GFP Constructs (A) Neural network—gene B0041.7a. (B) Labial sensilla (arrow)—gene C17E4.5. (C) Amphid neuron—gene T26E3.9. (D) Ring interneuron—gene H13N06.6 (intersects with WormBase annotation). (E) Amphid socket cells (arrow)—gene Y39D8C.1. (F) Pre-anal (short arrow) and lumbar (long arrow) ganglion—gene C47A10.6 (intersects with WormBase annotation). (G) Phasmid neurons—gene W01A11.2. (H) PVT interneuron—gene M03F4.3 (intersects with WormBase annotation).

Of the 1,886 genes with analyzable expression, only one in five was found to be tissue specific and only a very few were found to be cell specific (Figure 5; Table 1). Cell-specific promoters were found in a few special cases, as in the excretory cell in which we identified six 5′ regulatory regions that drove expression in only this cell (Figure 5). In another example, we found four specific cases of 5′ sequences limiting expression to the head mesodermal cell (Figure 5). In this study, we did not find any examples where individual cells belonging to a larger tissue group such as body-wall muscle, or hypodermis, or the intestine expressed by themselves. Tissue-specific GFP expression accounted for 20% of our samples, and all major tissues in this organism are represented in our dataset (Figure 6; Table 1). Of the 414 tissue-specific regulatory regions identified, the majority are expressed exclusively either in neural (l55; Figure 7 displays several examples of the complexity of the nervous system) or intestinal tissue (136). Other tissues or cell groupings that exhibited exclusive expression include the pharynx (40), body-wall muscle (18), reproductive system (14), hypodermis (10), hypodermal seam cells (8), pharyngeal gland cells (4), and the arcade cells (2).

**Figure 8:**
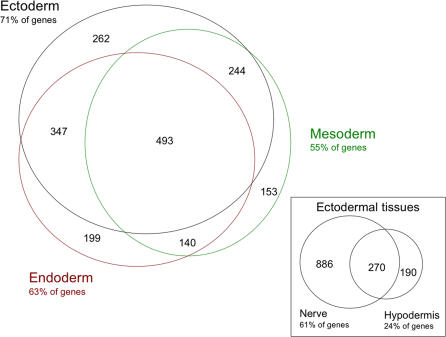
The Primordial Germ Layers' Main Sphere of Influence: Ectoderm (Neurons and Hypodermis), Mesoderm (Muscle), and Endoderm (Intestine) From the data, we see a fairly even distribution of expression between the germ layers: 71% of the 5′ DNA sequences express in ectoderm, 63% in endoderm, 55% in mesoderm, and about half from each germ layer contributes to the intersection of the three germ layers. Within the ectoderm (see inset), we see that there is a preponderance of neural-specific expression (61%) relative to hypodermal-specific expression (14%). There are very few hypodermal 5′ DNA sequences that do not also express in either the nerve, muscle, or intestine.

**Figure 9:**
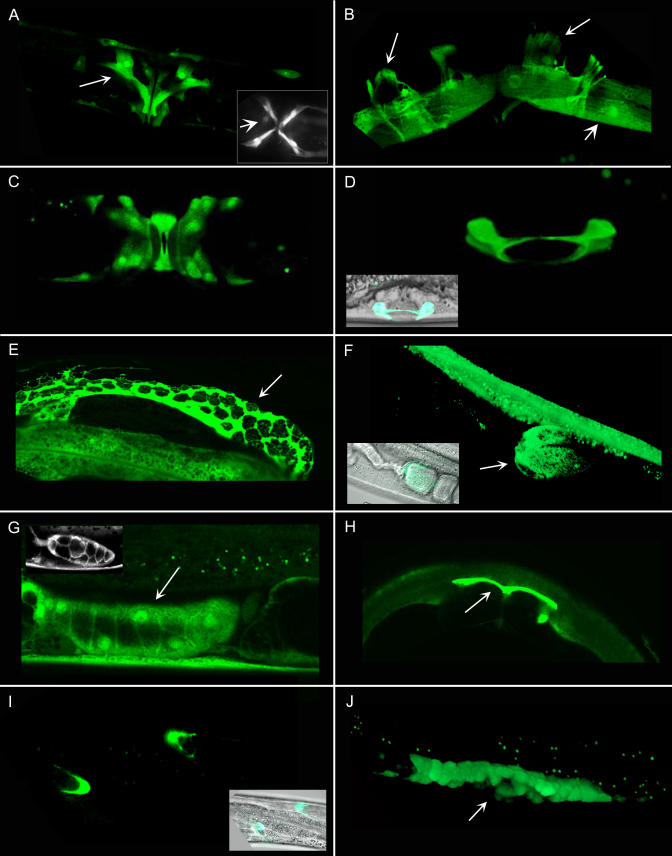
A Portrait of C. elegans Reproductive Tissue Expression Patterns, Driven by 5′ DNA::GFP Constructs (A) Vulval muscles (lateral view: long arrow, ventral view: short arrow)—gene F54A5.3a (inset F33H2.3). (B) Uterine muscles (long arrows) and body wall muscle (short arrow)—gene F10G12.5. (C) Vulva—gene Y47G6A.7. (D) VulA cell—gene F02D8.2. Inset shows VulA cel relative to other vulva cells. (E) Gonad sheath cells (arrow) and gut—gene C30F12.1. (F) Gonadal sheath cell pair 5 (arrow) and gut—gene T60A3A.9. Inset shows proximal gonadal sheath cells. (G) Spermatheca (arrow)—gene Y43B11AR.4 (inset F40F11.2). (H) Dorsal uterine cell (arrow)—gene C30F12.1. (I) Distal tip cells—gene C44B12.2. Inset shows distal tip cells at end of gonad. (J) Developing uterus—gene Y47G6A.7 (arrow indicates location of developing vulva).

When we examine the remaining genes, we observe that 321 of these regulatory regions drive expression in only two tissues. In the majority of these examples (72%), one of the two tissues involved is neural. We detected no bias for specific combinations of tissues or specific exclusions (Figure 8). Co-expression in nerve and muscle (604 examples), nerve and intestine (698 examples), or intestine and muscle (532 examples) are all roughly equivalent, with relatively little contribution from hypodermal expression. Cell- and tissue-specific regulatory regions clearly account for a minority of our expression examples, because the majority of 5′ regulatory regions we have analyzed, 1,151 (61%), drive expression in several tissues. (This is reflected in the Venn diagram of Figure 8 in which 493 examples express in at least three of the tissues being examined). A portion of this last group may represent ubiquitous expression, but it is not always possible to conclude that every cell expresses GFP. Widespread expression in an animal can make it extremely difficult to detect expression in each cell. In these cases, mosaicism of expression, rather than a hindrance, can be helpful. Figure 9 illustrates how mosaic expression can be used to advantage to obtain images of structures within the somatic gonad (Figure 9A, 9B, 9C, 9E, and 9G) and individual cells of the gonad (Figure 9D, 9F, 9H, and 9I). All of the examples in this figure are for genes that show expression in many different cells and tissues (see database at http://gfpweb.aecom.yu.edu/index for details on each gene.)

### Using Expression Data to Mine for Minimal Promoters and Conserved Motifs

**Figure 10:**
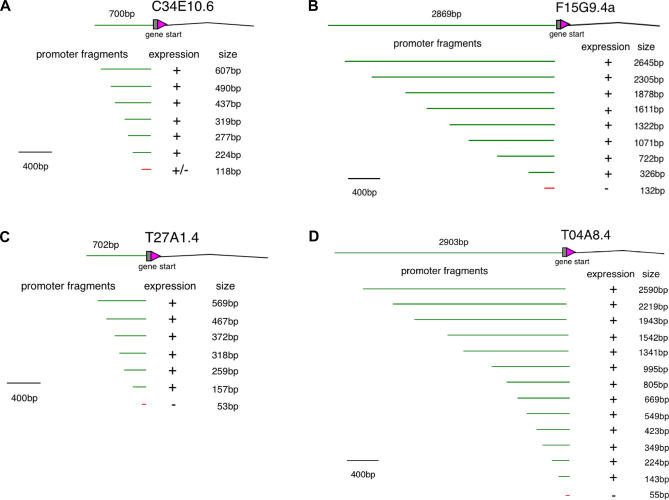
Serial Deletions Series for the Promoter Regions Used To Determine the Minimal Sequence Required to Drive GFP Expression in Muscle (A) C34E10.6 promoter length 700–118 bp drives expression in muscle and neural tissue. (B) F15G9.4a promoter length 2,869–326 bp drives expression in body wall muscle; at 132 bp, all expression was lost. (C) T27A1.4 promoter length 702–157 bp drives expression only in muscle; at 53 bp, all expression was lost. (D) T04A8.4 promoter length 2,903–995 bp drives expression in pharyngeal, vulval, and body wall muscles; promoter length 805–143 bp fails to drive expression in pharyngeal muscle; at 55 bp, all muscle had lost expression.

A large source of regulatory sequences and expression data permits investigation of regulatory sequences required to drive expression in a specific cell or tissue type. We use muscle as an example of how this dataset can be employed. We first identified several 5′ sequences capable of driving expression of GFP in body-wall muscle. We next took a subset of these sequences (four) and mapped out the region responsible for muscle expression by constructing a deletion series (Table S3 lists primers used for this deletion series). These deletion constructs determined the minimal 5′ DNA sequence required to drive muscle expression. The four gene promoters analyzed in our study were those of F15G9.4a, C34E10.6, T04A8.4, and T27A1.4 (Figure 10). From the deletion series, we found that the minimal length required for muscle expression varied between the promoters, the longest being 326 bp (Figure 10B), whereas the shortest was only 143 bp (Figure 10D). When compared to each other, except for T27A1.5 which contains an E box consensus sequence, the minimal promoters were found to contain neither any shared motifs nor any of the previously identified muscle motifs [22,23] (unpublished data).

## Discussion

Within this database, there are representatives of many of the expression patterns that are possible in this organism. We have identified 5′ DNA sequences that drive expression in single cells, in single tissues, in multiple tissues, and in all tissues. The dataset is large enough so that one can make some general statements about patterns of expression in this organism. One conclusion from these data is that expression within only a single cell using extant 5′ sequences is rare. The examples that exist in our dataset are usually examples in which a single cell is equivalent to a tissue, as in the case of the excretory cell. However, tissue-specific 5′ regulatory regions are abundant. We found many examples of expression limited to a single tissue, and this included such tissues as the intestine, muscle, and the nervous system, the primary tissues arising from the three primordial germ layers, of endoderm, mesoderm, and ectoderm (Figure 8). There are also expression patterns that represent subsets of these tissues and expression patterns that are specific to organs or specialized groupings of cells within these broader tissue categories, for example, expression in the pharynx, but not other muscle, or expression in the amphids/phasmids, but not other cells of the nervous system. We also identified 5′ regulatory regions that are not limited to regulating expression in a single tissue, but may include two or more tissues and even cells from several tissues. We also identified several 5′ sequences that apparently permit ubiquitous expression (at least 1%). Finally, we observed 516 5′ regulatory regions that did not exhibit any detectable expression. Although there are several trivial explanations for why these regions do not promote expression, there is also the possibility that these are conditional promoters. Several laboratories have requested these strains to test for expression in different genetic (male vs. hermaphrodite) or environmental backgrounds. So far, none have been shown to be conditional promoters.

The paucity of 5′ regulatory regions that drive expression in a single cell is perhaps disappointing, but it should not be a surprising result. At least one quarter of the genome is expressed in any particular tissue or cell type ([1], unpublished data; http://tock.bcgsc.ca/cgi-bin/sage170) which, as we observed, suggests even tissue-specific control regions will be relatively infrequent. To identify regulatory regions that drive expression in only single cells in this organism may require other approaches. In our experience, single unique genes predominantly express in multiple cell types. One possible way to identify cell-specific control elements may be to focus attention on gene families or alternative splice forms of a single gene. The seven transmembrane domain and guanyl cyclase gene families of receptors are excellent examples of gene families in which isoforms are specific for separate sensory neurons [24,25]. In this study, we did not focus on gene families, but it may be the approach one should take if the objective is to identify cell-specific markers.

The database should not be viewed as the final arbiter of complete expression for any specific gene. As we have only included DNA 5′ of a particular ORF, we may not have the complete “promoter” or all possible “enhancer” elements that impinge on the regulation and expression of this gene when located at its proper location within the chromosome. Our analysis misses any downstream, intronic, or more than 3-kb upstream elements important for proper gene expression. Because of this, a gene's complete expression pattern may differ from that observed using our reporter constructs. As well, 85% of the strains we examined had concatamer arrays with multiple copies of the regulatory region of the gene. This led to mosaic expression when the concatamer was lost, which meant that we had to be sure to examine several animals to ensure that we described all expression patterns possible using this stretch of DNA as a control element. Stably inherited constructs were made for about 15% of the samples, including those from which we desired to make an embryo 4-D recording. Note that the aforementioned caveats are not unusual, as most single gene studies reported in most C. elegans publications work with the same limitations (see expression report summaries in WormBase). If one uses reproducibility as a benchmark, then the data reported here are quite reliable. First, we compared our GFP expression data to expression data using SAGE to detect tissue-specific transcripts and found that about 70% of the genes found expressed in a particular tissue by our GFP reporter assay were also detected using SAGE analysis. We also included in our analysis several genes whose expression was previously characterized, either by GFP promoter constructs or protein fusions or by antibodies. For more than 80% of the previously characterized genes we examined, the expression pattern is in good agreement with published observations. In some cases, we observed a wider range of expression, and in some cases, we observed less. In less than 10% of cases, our observations were completely at odds with what has previously been published. Due to the possible differences in size of 5′ promoter regions, differences in concatamer arrays, or even entirely different methodologies, these discrepancies should not be too surprising. In regard to this benchmark set of genes, often it is not clear whether our observations are the correct ones, or whether previous observations are correct, or if neither reflect the full range of expression of the gene in question. What we are certain of is that the annotation of tissue and cell identity is correct in our study. We have called upon experts within the C. elegans community and the staff of WormAtlas in every instance in which there was a question of cell identity. If cell identity could still not be resolved, this was indicated in the annotation. If there are errors, they are errors of omission, not errors of commission.

With almost 2,000 expression profiles, the database is an excellent resource for examining the expression profile of a previously uncharacterized gene, even with the caveats stated above. However, we do not feel this is the only possible use of the data. The data reflect expression from less than 5 Mb of DNA, less than 5% of the genome of this organism, and yet we see expression in almost every tissue and cell type in the organism. We think this is fertile ground for researchers interested in identifying motifs regulating gene expression. In many cases, the DNA segment regulating precise cellular and temporal expression is considerably shorter than our maximum size fragments of 3 kb. The ability to search this database for short DNA sequences controlling specific expression patterns should make it easier to identify transcription factor binding sites for a particular organ, tissue, or cell type.

Our survey of a few regions determining expression within muscle serves as a case study. We first identified several genes expressed within body-wall muscle. We then picked a subset of 5′ regions and did promoter deletions in order to map essential sites for muscle expression. Curiously, we did not find any single motif, but in fact, found several potential sequences that each could direct expression in muscle (unpublished data). The implication of these observations is that different 5′ sequences can lead to expression in the same tissue, in this case muscle, and we suspect this multiplicity of transcriptional control regions may occur in other tissues as well. This adds a level of complexity to gene regulation that many researchers fail to take into consideration. Our findings of multiple different sequences controlling muscle expression are similar to results reported previously [22,23], but the sequences we have identified are different from those reported in these earlier studies. Even though a MyoD homolog *(hlh-1)* [26–28] is expressed in C. elegans muscle, it does not seem to be the major transcription factor, because no MyoD binding site has been found in three of four control regions we analyzed. Recently, it has been shown that MyoD acts as part of a trio of transcription factors to regulate muscle differentiation in C. elegans [29].

Many of the genes in this expression database have human orthologs, and for a number of these genes, these expression data are the first indication of where these genes may be expressed in humans. We think this is an important resource to help direct studies of these genes in mammals. Considering the complexity of the mammalian nervous system, any gene that we can identify in a particular subset of neurons may be especially useful. Another use of the database has been to confirm an expression profile of a specific gene identified by other methods. Studies of adult intestine and ciliated neurons have used the GFP strains described in this database as confirmation of tissue-specific expression of genes identified by SAGE tags found in these tissues [30,31].

The GFP constructs described in this study are relatively easy to make and thus lend themselves to a high-throughput strategy. The PCR-stitching strategy we used [15] has proven robust and efficient. This approach has at least one advantage over the newly developed “promoterome” [12], which is that significantly larger 5′ regions can be used for stitching when necessary. Many regulatory regions are close to the ATG start site, as shown for the four genes we analyzed for muscle expression (Figure 10), but this is not always the case. A further complication with plasmids is that they often contain cryptic promoter elements, which one can avoid by using the PCR-stitching approach. The use of freely segregating concatamer arrays for this study had three implications. It appears from a comparison of GFP expression with low tag-frequency SAGE data that concatamer arrays of GFP may be a sensitive tool for detecting genes with a low level of transcription. We also demonstrated that mosaicism due to loss of the array often led to expression in small groups of cells or single cells, and thus allowed us to obtain a detailed image of these cells. This has been an invaluable aid to the WormAtlas project (http://www.wormatlas.org/). On the other hand, an unfortunate consequence of using a concatamer array was that it excluded us from recording germline expression and thus monitoring the maternal contribution to early development. Germline silencing of genes is well documented [21], and this silencing led to us not detecting germline expression in any of the genes we tested. It also meant that we could not detect expression in the early embryo (before 50 cells) in most cases.

In addition to the approaches described in this study, other approaches to monitor gene expression will be required if we are to monitor gene expression for the whole genome throughout all of development. The technique of homologous recombination in E. coli called recombineering [32–36] is a promising approach because it allows the modification and manipulation of large genomic clones. Larger DNA clones would remove some of the doubt about whether all control elements for transcription regulation are included. Recombineering in bacteria to construct GFP::protein fusions using fosmids with 35- to 40-kb DNA inserts should cover all control elements for most genes in C. elegans. We have built a C. elegans fosmid library, and clones from this library are being used for recombineering (http://elegans.bcgsc.bc.ca/perl/fosmid/CloneSearch) ([33] and unpublished data). If these GFP-engineered fosmids are introduced to the worm using a Biolistic gun [37,38], there is a higher probability of generating a transformed animal with a single or low copy number level of the gene. This should allow expression in the germline and the early embryo of any gene in which these are the normal sites of expression. Coupling these strategies to the newer methods of lineaging early cell division [39] should cover the stages in development overlooked in our study.

## Materials and Methods

### Identifying gene targets.

Our list of target genes was based on the 4,367 C. elegans proteins identified from a comparison of C. elegans and human predicted proteomes with InParanoid [16] (http://inparanoid.sbc.su.se), Most of the genome annotations used in the selection of our list of target genes were obtained from WormBase [40,41] (http://www.wormbase.org). The list was filtered to remove rRNA genes and genes with SL2 trans-splice acceptor sites, which are associated with operons [42,43]. Also removed were genes with characterized mRNAs, an indication that the gene was already well studied. Preference was given to genes with EST-confirmed 5′ ends and those identified as embryonically expressed in Intronerator [44]. We kept genes for which other researchers have constructed reporter fusions as a control set for our study. Our final set of targets consisted of a gene pool enriched for, although not exclusive to, human orthologs with unknown function.

### Primer design and construction of GFP fusions.

The promoter::GFP fusion constructs were generated using the PCR stitching method from Hobert [15]. The PCR experiments were designed to capture putative 5′ DNA regions by amplifying about 3 kb of genomic DNA sequence immediately upstream of the predicted ATG initiator site. When an upstream gene was within 3 kb, the size of the amplicon was adjusted downward. We set the maximum primer length to be 25 nucleotides, and in order to eliminate false-positive PCR products, we designed a nested primer immediately downstream from the most 5′ primer for the second-round reaction. Where the primer encompassed the ATG initiator site, the G was mutated to a C, to ensure there was only one start codon in the promoter::GFP fusion.

Early PCR experiments were designed semimanually with the aid of primer3 [45]. To facilitate scale-up, we used Perl and AcePerl [46] to extract C. elegans genomic DNA sequence, and annotations from WormBase to tie them together with the primer design and validation programs primer3 and e-PCR [47]. An interactive version of the GFP primer design program is available at http://elegans.bcgsc.bc.ca/promoter_primers.

We used pPD95.67 variant S65C (developed by Dr. Andrew Fire, Carnegie Institution, http://www.addgene.org/pgvec1?f=c&cmd=showcol&colid=1) as our GFP source because it contains a GFP-cassette and a region that has sequence overlap with the 3′ primer, thus allowing for PCR stitching. 5′ DNA regions from target genes were amplified from C. elegans N2 (Bristol) genomic DNA.

DNA amplification mixtures consisted of Mix 1: 0.5-μl dNTP (10 mM), 1-μl N2 genomic DNA, 21.5-μl double-distilled H_2_O (ddH_2_O), 5′ and 3′ primers (1 μl of 12.5 μM each); and Mix 2: 0.75-μl Long Taq (Expand Long Template PCR System made by Roche Diagnostics, http://www.roche.com), 5-μl 10× Long PCR buffer (#2 from kit), 19.25-μl ddH_2_O. Mix 1 and Mix 2 were combined, and PCR was carried out for 30 cycles under the following conditions. Step 1: (1 cycle) 94 °C for 1 min. Step 2: (30 cycles) denaturation at 94 °C for 10 s, anneal at 56 °C for 30 s, and elongation at 68 °C for 2.5 min (depending on amplification fragment size). Step 3: 68 °C for 5.5 min. Stitched PCR product was constructed as follows: Mix 3: 5′ and 3′ primers (1 μl of 12.5 μM); 0.5-μl 5′ regulatory DNA PCR product, 0.5-μl GFP PCR product, 1.5-μl dNTP 10 mM, 21-μl ddH_2_O, and Mix 4: 5-μl 10× Long PCR buffer, 20-μl ddH_2_0. Mix 3 and Mix 4 were combined. PCR was done as follows. Step 1: (1 cycle) 94 °C for 1 min. Step 2: (18 cycles) denaturation at 94 °C for 10 s, anneal at 56 °C for 30 s, and elongation at 68 °C for 2.5 min. Step 3: (10 cycles) 94 °C for 10 s, 56 °C for 30 s, and 68 °C for 2.5 min (increased by 10 s each cycle). The PCR product was stored at 4 °C.

### Constructing transgenic animals containing GFP fusions.

Nematode strain maintenance and culture were carried out as described by Brenner [48]. Strains were maintained at 15 °C on OP50 plates unless otherwise specified. Strains used include *dpy-5(e907)* and wild-type N2 Bristol [48].

At the beginning of the project, we injected a number of strains, with-gel purified DNA, and came to a similar conclusion as Hobert [15], that gel purifying DNA for injection did not significantly change the results.

Transgenic worms were generated by a modification of the method described by Mello et al. [49]. 5′ regulatory DNA::GFP constructs and *dpy-5*(+) plasmid (pCeh-361) (kindly provided by C. Thacker and A. Rose; [50]) were used to construct transgenic strains. Transformants were identified by rescue of the *dpy-5* mutant phenotype. The 5′ regulatory DNA::GFP fusions were co-injected with wild-type *dpy-5* plasmid DNA into P_0_ *Dpy-5(e907)* gonads using one of these systems: a Olympus BH2-HLSK with a Leitz Westlab injection needle manipulator, or a Zeiss 47 3016 microscope (Carl Zeiss, http://www.zeiss.com) with a Leitz Westlab injection needle manipulator (http://www.leitz.org/leitz_english/index.html), or a MINJ-7 microinjection system with an Olympus CK40 microscope from Tritech Reseach (http://www.tritechresearch.com). Injection mixture included ddH_2_O, 10× TE, *dpy-5* plasmid (*pCeh361,* concentration 5–80 ng/μl), and 5′ regulatory DNA::GFP fusion construct (concentration 50 ng/μl). A total of 1 nl of the final mix (80–90 ng/μl pCeh361 and 5–20 ng/μl DNA::GFP fusion) was microinjected into P_0_ worms using 1.0-mm, 6” filamented capillary tubes from World Precision Instruments (http://www.wpiinc.com) pulled on a Sutter P-97 needle puller. P_0_ worms were set up for microinjection on agarose pads (2%–3% agarose flattened on cover slips) in either mineral oil (Sigma) or in halocarbon oil #700 grade (Lab Scientific, http://www.labscientific.com). An injection set consists of 25–50 P_0_ worms injected with a given 5′ regulatory DNA::GFP construct. Wild-type F_1_s were set up individually and their progeny were screened for wild-type animals in the F_2_ generation. One or two lines yielding at least 30% wild-type progeny were maintained as transformed stocks. For promoter analysis, DNA was injected at 40–60 ng/μl for both subcloned constructs and PCR fusions, using *rol-6* as an injection marker.

To determine the size of the concatemeric arrays in vivo, we used quantitative PCR to estimate the copy number of the 5′ DNA::GFP constructs and plasmids in 20 different transgenic strains. We estimated that there were about 5–10 copies of promotor::GFP and 100–600 copies of the *dpy-5* plasmid in the heritable arrays, which was sufficient for the sensitivity of our GFP assay.

We constructed chromosomal integrant strains for a subset of the GFP constructs (1%) using a modified version of M. Koelle's method (http://info.med.yale.edu/mbb/koelle/). Young adult transgenic (wild-type) P_0_ hermaphrodites were treated with low-dose X-ray irradiation (1,500 R). After 1 h, the P_0_ animals were transferred to 90-mm OP50 plates—one P_0_ worm/plate for 12 plates for each strain. The P_0_ animals were allowed to lay eggs for 18–24 h and then were removed in order to limit the number of F_1_s laid. Seven days later, mid to late larval wild-type F_2_ animals were picked and set up (one/plate, 12 from each of the 90-mm plates) at room temperature (20–22 °C). Four to 5 d later, the F_2_ plates were screened for the absence of Dpy-5 animals, indicating stable inheritance of the array.

Strains intended for embryo recordings were outcrossed using an *unc-32* marker. P_0_ GFP-expressing hermaphrodites were crossed with N2 males, F_1_ GFP males were crossed with *unc-32* hermaphrodites, and then F_2_ and F_3_ GFP hermaphrodites were individually plated. Lastly, the F_4_ populations were screened for exclusively wild-type animals. Outcrossing was done at 15 °C.

### In vivo analysis and imaging of GFP-expressing animals.

General classification and imaging of GFP expression was done initially with a low-power GFP dissecting microscope (Zeiss stereomicroscope fitted with Kramer epifluorescence), before moving to either a Zeiss Axioplan or a Zeiss Axiophot microscope. Images were captured using a digital camera (QICAM; QImaging, http://www.qimaging.com/products/cameras/scientific/) and QCapture software. This was the first pass, where we determined the developmental stage, tissues, and, where possible, the individual cells expressing GFP. Both stable and unstable strains were evaluated on expression pattern complexity and frequency of occurrence. Unusual or complicated expression patterns, or neural expression, would undergo further analysis using an inverted Zeiss Axiovert LSM 5 confocal microscope equipped with epifluorescence, Nomarski optics, and LSM 5 Pascal software. If we detected pre-comma nonubiquitous expression, strains were put in queue for stabilization and/or outcrossing, and 4-D recording and analysis. The results of all analyses, excepting of the embryos, were curated by hand and uploaded to the project Web site (http://gfpweb.aecom.yu.edu/index) and WormBase (http://www.wormbase.org), and the strains were sent to the stock centre (http://www.cbs.umn.edu/CGC/CGChomepage.htm) and are available by request from R. Johnsen (bjohnsen@gene.mbb.sfu.ca) (see expression pipeline in Figure 1).

The images and movies were processed using Adobe PhotoShop 7.0 (http://www.adobe.com) and the LSM 5 Pascal volume-rendering software. Single images were normalized and placed into image panels before exporting to the public domain. Movies were obtained from *Z*-stacks comprised of 20–60 *.lsm images, taken 0.5–1-μm intervals apart, the specifics of which were dependant on the age of the worm, the tissue of interest, and the intensity of the GFP. These stacks were then optionally volume-rendered and/or converted into QuickTime movies, normalized, and exported to the Web site.

### Analysis of embryonic expression using 4-D microscopy.

In some cases, embryonic expression was determined without difficulty. However, in many cases, the patterns were determined to be too complex, and it was deemed necessary to have a 4-D recording and to lineage the embryo. Embryos for 4-D analysis were obtained from gravid hermaphrodites. Two embryos at the 2–4 cell stage, were transferred to a 5% agar pad and manipulated into adjacent positions with the same orientation. Detailed expression patterns and gene activation in the embryos were captured with live, two-channel, four-dimensional microscopy, on a Zeiss Axioplan microscope. The fourth dimension being time, *Z*-stacks (25 Z-images) of developing embryos were recorded at 25 °C using Nomarski microscopy every 30–45 s over a 7-h time course. Interspersed with the normal *Z*-stacks, we recorded several *Z*-stacks of GFP fluorescence in specific cells, which were then mapped and identified relative to the Nomarski images. Software that supports this type of microscopy recording and analysis has been developed [19,51–53]. We used programs derived from the study by Schnabel et al. [19] and the program Simi Biocell to lineage the embryos [19]. The data have not been posted to the Web site, but are available from the authors.

### An interactive GFP expression Web site.

All strain data are in a mySQL database. All primer designs relative to genes and all annotation of genes on the Web site are based on WormBase version 140. The functionality of the Web site is based on perl/CGI and perl modules for the queries, which provide the user with three formats for accessing the data: (1) the display of all strains and data for browsing, (2) the selection of specific genes and the information the user wants to see for each gene, and (3) a gene search, based on tissue expression pattern. All of the data can be downloaded in .tab or .csv format from WormAtlas (http://gfpweb.aecom.yu.edu/index). The data are also available at WormBase (http://www.wormbase.org).

## Supporting Information

Figure S1 Comparison of Biological Process GO Annotation between the GFP Gene Set and across the Whole Genome (1.0 MB TIF)

Table S1 A Subset of Our Positive Controls Compared with Prior Expression Annotations in WormBase The annotations for the positive controls in both our dataset and WormBase were manually compared to determine the degree to which our results intersected with other labs. The primary techniques listed in WormBase were both transcriptional and translational GFP fusions, LacZ reporters, and antibody staining.
(54 KB DOC)

Table S2 Confirmation of 5′ DNA::GFP Fusion Sensitivity in C. elegans by Comparison with Single SAGE Tags There are 232 single-tag genes for which we detect a GFP signal.
(111 KB DOC)

Table S3 Primers Used in PCR Fusion Constructs for Promoter Analysis of Muscle-Expressing 5′ DNA Sequences (80 KB DOC)

Video S1 C. elegans H13N06.6 Promoter::GFP Expression (a Gene Related to Norepinephrine Deficiency in Humans) in the Gonad Sheath Cells, Spermatheca, and Uterus of the Adult Worm (2.1 MB MOV)

## Abbreviations

GFP: green fluorescent protein
SAGE: Serial Analysis of Gene Expression
