# Supplementary material for: High-Throughput In Vivo Analysis of Gene Expression in Caenorhabditis elegans
Source: PLoS Biol. 2007 Sep 11;5(9):e237. doi: 10.1371/journal.pbio.0050237 (PMC1971126; doi:10.1371/journal.pbio.0050237)
Supplement: Supplementary file 1 [file pbio.0050237.st001.doc]

|  | **Genes (locus)** | | | | |
| --- | --- | --- | --- | --- | --- |
| **similar pattern *** | AH6.10 (sra-6) | B0280.1a | B0304.1a (hlh-1) | B0336.2 (arf-1) | C01B7.6 (rpm-1) |
|  | C03B8.4 (kin-13) | C05E4.1 (srp-2) | C06C6.5a (nhr-50) | C09E7.3 (oig-1) | C09E10.2a (dgk-1) |
|  | C10H11.9 (let-502) | C17H12.14 (vha-8) | C29F9.7 (pat-4) | C32E12.3 (osr-1) | C52E12.2 (unc-104) |
|  | E02H4.1 (del-1) | E03G2.3 (mec-4) | F01G12.5a (let-2) | F09C3.1 (pes-7) | F10F2.4 |
|  | F11C3.3 (unc-54) | F14F3.1a (vab-3) | F15D3.1a (dys-1) | F28F9.1 (zag-1) | F42G10.2 (mkk-4) |
|  | F44F4.5 (sra-10) | F44F4.13 (sra-11) | F54D7.4 (zig-7) | F55C7.7a (unc-73) | F49E12.5 (sra-13) |
|  | K03A11.3 (ceh-28) | K12G11.2 (sulp-5) | T02C5.5b (unc-2) | T08G11.1b | T27B1.1 (osm-1) |
|  | R05F9.1a | R05H5.6 (srb-6) | Y47D3A.6b (tra-1) | ZK455.7 (pgp-3) | ZK524.2a (unc-13) |
| **some overlap in patterns ^** | C05B10.1 (lip-1) | C07H4.2 (clh-5) | C44C1.4 | F25H8.3 (gon-1) | F39G3.7 (prx-6) |
| **dissimilar pattern ~** | C02B8.4 (hlh-8) | C02H7.3 (aex-3) | C07D10.4 (nas-7) | C09D8.1a (ptp-3) | D2085.5 |

Supplementary Table 1 A subset of our Positive controls compared with prior expression annotations in WormBase.

* >70% agreement between our controls and WormBase

^ a few tissues/cells in agreement; one source often has limited or ubiquitous annotation

~ no agreement between our controls and Wormbase
