# Supplementary material for: High-Throughput In Vivo Analysis of Gene Expression in Caenorhabditis elegans
Source: PLoS Biol. 2007 Sep 11;5(9):e237. doi: 10.1371/journal.pbio.0050237 (PMC1971126; doi:10.1371/journal.pbio.0050237)
Supplement: Supplementary file 2 [file pbio.0050237.st002.doc]

Supplementary Table 2 Confirmation of 5’DNA::GFP fusion sensitivity in *C. elegans* by comparison with single SAGE tags.

| **Embryo** | | **Neural tissues** | **Hypodermis** | **Gut** | | **Body wall muscle** |
| --- | --- | --- | --- | --- | --- | --- |
| B0218.8 | H20J18.1a | B0024.14a | F28F8.6 | AC7.2a | C44C1.2 | B0272.3 |
| B0272.3 | H28G03.6 | B0252.2 | B0464.5a | B0034.3a | C44H4.4 | C18F10.7a |
| B0414.2 | JC8.10a | B0285.3 | C04G6.3 | B0218.8 | C46C11.1 | C27A7.4 |
| B0414.5 | JC8.10b | B0336.4 | C18F10.7a | B0286.4c | C46H11.11b | C30C11.2 |
| B0464.5a | K03B8.5 | B0361.6 | C25A1.5 | B0334.4 | C49H3.10 | C41D11.2 |
| C02F12.5 | K04H4.6a | C01F1.6 | C30F12.2 | B0350.2f | C50D2.2 | C56G2.3 |
| C02H7.1 | K06A1.4 | C06E8.3a | C38C10.5a | B0464.5a | C52E12.3 | F08B12.3b |
| C02H7.3a | K11G12.1 | C07E3.5 | D2085.3 | B0496.8 | C53A5.3 | F11G11.7 |
| C03D6.5 | R07B7.1 | C09E10.2a | F09C3.1 | B0547.1 | CD4.2 | F28F8.6 |
| C04A11.3 | R10E11.1a | C09F5.2 | F22E10.1 | C01B7.4 | E03G2.3 | F42E11.1 |
| C04C3.5 | T01E8.2 | C09G4.1 | F36F2.5 | C01G10.10 | F08F3.3 | F43G9.3 |
| C04G6.3 | T04C9.6 | C10C6.5 | F52D10.2 | C02C6.1a | F10D2.9 | F43G9.5 |
| C05C10.6a | T09E8.2 | C11H1.3 | F53B1.2 | C03D6.3 | F21G4.2 | F49C12.8 |
| C06C6.5a | T15H9.3 | C13D9.1 | F56H11.4 | C04A11.3 | F28F9.1 | F56H11.1b |
| C06E7.1a | T21E12.4 | C13F10.7 | F57C12.1 | C05C8.2 | F35G12.8 | F59B10.1 |
| C38D4.8 | T23G11.2 | C15C7.2 | K11G12.1 | C05D9.1 | F36F2.5 | K02A4.1 |
| C50C3.9 | T27B1.1 | C17G10.2 | M04C9.5 | C06C6.5a | F37A4.8 | K08F8.6 |
| C53A5.3 | W04B5.5 | C17H12.13 | R07B7.11 | C08B6.8 | F44B9.3a | K12C11.4 |
| F08B12.3b | Y105E8A.5 | C18E9.6 | R11A5.1b | C09F5.2 | F47D12.4b | M01G5.3 |
| F08F8.3a | Y111B2A.18 | C30F12.2 | R151.2b | C10H11.9 | F54D8.3a | R03E1.1 |
| F09E8.6 | Y39A1B.2 | C33A11.4 | T01B10.2 | C13C4.5 | F55H2.1 | R06F6.5a |
| F35G12.6 | Y50D7A.6 | C35B8.2 | T01C8.5 | C18F10.8 | F57C12.4 | R11F4.1 |
| F36F2.5 | ZC101.2e | C38H2.1 | T05C12.10 | C23G10.8 | JC8.10a | R13A5.1a |
| F42E11.1 | ZK112.2 | C41D11.2 | T09F3.2 | C24B9.2 | K02A4.2 | T08G11.1b |
| F52C9.8a | ZK455.7 | C42D8.3 | T13B5.1 | C24G6.7 | K06H7.4 | T21E12.4 |
| F54D7.4 | ZK512.6 | C44H4.5 | Y108G3AL.1 | C26E6.4 | K07A9.2 | Y110A7A.3 |
| F54D8.3a | ZK856.1 | C46C11.1 | Y18D10A.13 | C27A2.6 | K08E3.6 |  |
| F55C7.7a | ZK896.8 | F14D12.4a | Y18D10A.6a | C30C11.2 | R07E4.4 |  |
|  |  | F16B3.1 | Y39A3CR.3 | C32E8.9 | R11A5.1b |  |
|  |  | F29F11.6 | Y57G11C.24c | C33H5.18a | R13F6.3 |  |
|  |  | F33H2.1 | ZK177.8a | C34B2.6 | T09E8.2 |  |
|  |  | F36F2.5 | ZK856.1 | C34G6.1 | T21E12.4 |  |
|  |  | F48E3.1a |  | C34H4.3 | W02A2.1 |  |
|  |  | F54D8.3a |  | C38C10.2 | Y108G3AL.1 |  |
|  |  | JC8.10a |  | C38H2.2 | Y48A6A.1 |  |
|  |  | JC8.10b |  |  |  |  |
|  |  | K11G12.1 |  |  |  |  |
|  |  | T04C9.6 |  |  |  |  |
|  |  | Y48A6A.1 |  |  |  |  |
|  |  | Y95B8A.1 |  |  |  |  |
|  |  | ZC247.3 |  |  |  |  |
|  |  | ZK524.2a |  |  |  |  |
|  |  | ZK54.2 |  |  |  |  |
