# Supplementary material for: High-Throughput In Vivo Analysis of Gene Expression in Caenorhabditis elegans
Source: PLoS Biol. 2007 Sep 11;5(9):e237. doi: 10.1371/journal.pbio.0050237 (PMC1971126; doi:10.1371/journal.pbio.0050237)
Supplement: Supplementary file 3 [file pbio.0050237.st003.doc]

Supplementary Table 3. Primers used in PCR fusion constructs for promoter analysis of muscle-expressing 5’ DNA sequences.

| Gene | Name | Sequence | Note |
| --- | --- | --- | --- |
| T04A8.4 | 1A | 5’-TTCCATCTGAACCAACGAGTC-3’ | 5’ promoter primer of T04A8.4 |
|  | 1B | 5’-GGATTTTGAGCTGAAAGTGATTG-3’ | 3’ promoter primer of T04A8.4 |
|  | 1A1 | 5’-ACCCACGAGACGCCAGTT-3’ | 5' shorten promoter primers of T04A8.4 |
|  | 1A2 | 5’-TTGAAGAGCAGCCAATCC-3’ |  |
|  | 1A3 | 5’-TCCGCTGATAAGAACAAGTGAA-3’ |  |
|  | 1A4 | 5’-TCCGTAGAGCCCGTCCGT-3’ |  |
|  | 1A5 | 5’-GAACATTGACCACCCAGT-3’ |  |
|  | 1A6 | 5’-CAAGTCAATCAGTGGAAGAA-3’ |  |
|  | 1A7 | 5’-AATCAAGATTCAACCACTCATA-3’ |  |
|  | 1A8 | 5’-TTTTCCGACGCTTGATTT-3’ |  |
|  | 1A9 | 5’-CAGCCTTCCTCGCTGTTT-3’ |  |
|  | 1A10 | 5’-AAGTCTGGGAGTCGTTCC-3’ |  |
|  | 1A11 | 5’-TTCCATTTGCCAAATTAA-3’ |  |
|  | 1A12 | 5’-CAAGTATCAACCTTCCAGAACAA-3’ |  |
|  | 1A15 | 5’-TTTCTCTCTCTCTCTTTTCC-3’ |  |
|  | C1A12 | 5’-GCTCTAGAGCCAAGTATCAACCTTCCAGAACAA-3’ | Subcloning primers |
|  | C1A14 | 5’-GCTCTAGAGCTCATCTTTAGCTGCTCGT-3’ |  |
|  | C1B | 5’-CGGGATCCCGGGATTTTGAGCTGAAAGTGATTG -3’ |  |
| C34E10.6 | 2A | 5’-TGAATTCTGCACTGCAATCAC-3’ | 5’ promoter primer of C34E10.6 |
|  | 2B | 5’-TAACGAACGCGAAGCGATA-3’ | 3’ promoter primer of C34E10.6 |
|  | 2A1 | 5’-TCGAGGTGGCGTAGTTGA -3’ | 5' shorten promoter primers of C34E10.6 |
|  | 2A2 | 5’-ATTGAACGATAATTAAAGGC -3’ |  |
|  | 2A3 | 5’-AGGGCATAAAACACGATTTC-3’ |  |
|  | 2A4 | 5’-TCTTGCCAAAATGCAAGC-3’ |  |
|  | 2A41 | 5’-GTTGCGAGACCCGCTG-3’ |  |
|  | 2A5 | 5’-GTAAGCTAACCAAGCGAATT-3’ |  |
|  | 2A6 | 5’-AAGCACCGAACAATTTTG-3’ |  |
|  | 2CbA2 | 5’-ACCCTGAAACCGAAAATTC-3’ | 5' promoter primer of C34E10.6 ortholog in *Cbriggsae* |
|  | 2CbB2 | 5’-AGTCGACCTGCAGGCATGCAAGCTagcgatagtttgtacccctg-3’ | 3' promoter primer of C34E10.6 ortholog in *Cbriggsae* |
|  | C2A6 | 5’-GCTCTAGAGCAAGCACCGAACAATTTTG-3’ | Subcloning primers |
|  | C2B | 5’-CGGGATCCCGTAACGAACGCGAAGCGATA-3’ |  |
| F15G9.4a | 3A | 5’-ACAACGGAATGCCTGAAAAC-3’ | 5’ promoter primer of F15G9.4a |
|  | 3B | 5’-GACTCCGTCCGATTTTTGAA-3’ | 3’ promoter primer of F15G9.4a |
|  | 3A1 | 5’-CAAGCACTAAGCGGACAA-3’ | 5' shorten promoter primers of F15G9.4a |
|  | 3A2 | 5’-CGCTTTGTGGTTTCAGGT-3’ |  |
|  | 3A3 | 5’-ATTCAAACGGCAAAACGG-3’ |  |
|  | 3A4 | 5’-GTGGAGTTGGGCGTAAAG-3’ |  |
|  | 3A5 | 5’-TGATGGCCCGATACTGAA-3’ |  |
|  | 3A6 | 5’-GCGGCGTCCTATACTAAT-3’ |  |
|  | 3A7 | 5’-GCCCCGGGTTCCCGAGAC-3’ |  |
|  | 3A8 | 5’-CAATATCAATCCATCAAC-3’ |  |
|  | 3A9 | 5’-TTGCTAGCGTTCATGCTT-3’ |  |
| T27A1.4 | 9A | 5’-TTCCATCTGAACCAACGAGTC-3’ | 5’ promoter primer of T24A1.4 |
|  | 9B | 5’-GGATTTTGAGCTGAAAGTGATTG-3’ | 3’ promoter primer of T27A1.4 |
|  | 9A1 | 5’-GCTAAGGCTTATCTCCGCAGTC-3’ | 5' shorten promoter primers of T27A1.4 |
|  | 9A2 | 5’-TTGAGATTTTGAATTTCAATAGAATGAC-3’ |  |
|  | 9A3 | 5’-CTCGAAAATACTGCTCATTC-3’ |  |
|  | 9A31 | 5’-AATTTTCAGTTTTAAATGCC-3’ |  |
|  | 9A4 | 5’-ACGTTTTCCGGCTCAATT-3’ |  |
|  | 9A5 | 5’-CCGCGAGCTCTGAAGTTC-3’ |  |
|  | 9A6 | 5’-AAAAGCTTCTTCTTTTTACT-3 |  |
